# Antagonistic activity of PGPR isolated from the avocado (*Persea americana*) rhizosphere on *Phytophthora cinnamomi* and *Lasiodiplodia theobromae*

**DOI:** 10.3389/fpls.2026.1872396

**Published:** 2026-08-10

**Authors:** Richard A. Solórzano-Acosta, Kenyi R. Quispe

**Affiliations:** 1 Dirección de Servicios Estratégicos Agrarios, Instituto Nacional de Innovación Agraria (INIA), Lima, Peru; 2 Facultad de Ciencias Ambientales, Universidad Científica del Sur (UCSUR), Lima, Peru

**Keywords:** antifungal activity, disease suppression, rhizobacteria, rootstock microbiome, sustainable agriculture

## Abstract

Plant growth-promoting rhizobacteria (PGPR) have proven to be a sustainable alternative for managing plant pathogens. Developing inoculants with these PGPRs requires a rigorous selection of highly effective native bacterial isolates. In the present study, 29 bacterial isolates were obtained from the rhizosphere of ‘Zutano’ avocado (*Persea americana* Mill.), a rootstock widely used for the commercial cultivar ‘Hass’, from a commercial orchard on the northern coast of Peru. Two antagonistic isolates showing the highest *in vitro* inhibition capacity were selected to evaluate their efficacy against the pathogens *Phytophthora cinnamomi* and *Lasiodiplodia theobromae*, the causal agents of avocado root rot and dieback, respectively. Results demonstrated that *Bacillus subtilis* exhibited strong *in vitro* antagonistic activity against *P. cinnamomi* and *L. theobromae*, with 44% and 33.33% inhibition, respectively. Additionally, inoculation of *B. subtilis* in avocado plants infected with *P. cinnamomi* resulted in an estimated 90% root viability based on a visual disease severity scale, while increasing root dry weight and length by 116.58% and 44.59%, respectively. These results highlight the potential of *B. subtilis* for the biological control of avocado diseases caused by *P. cinnamomi*, while *Pseudomonas putida* showed promising *in vitro* antagonistic activity.

## 1 Introduction

According to the Food and Agriculture Organization of the United Nations (FAO), Peru is the fourth largest avocado producer in the world, with a production of 982,558.94 tons in an area of 63,078 ha, reaching an average national yield of 15.57 t ha^-1^ (FAO, 2023). Additionally, Peru reached USD 1.22 billion in Free On Board (FOB) export value in 2024 (SIEA, 2024; Martínez et al., 2015). However, diseases caused by *Phytophthora cinnamomi* and *Lasiodiplodia theobromae* pose a significant economic threat to avocado orchards (Solís-García et al., 2021). These pathogens are the primary cause of avocado plant mortality during the early growth years (Barrientos-Priego et al., 2007). *P. cinnamomi* is considered the most limiting phytopathological problem of the crop worldwide, due to its frequency, severity and high economic losses (Ramírez‐Gil et al., 2017). *L. theobromae* is recognized as one of the most aggressive pathogens because it is a pathogenic fungus of more than 500 species of plants, including avocado (*Persea americana* Mill.) (Rodríguez-Gálvez et al., 2025). Although detailed quantitative surveys of yield losses caused by *L. theobromae* are not available for most avocado-producing regions, estimates indicate that this pathogen is associated with stem-end rot disease in 82% of plots located in Peru (Rodríguez-Gálvez et al., 2021).

*Phytophthora cinnamomi* is the pathogen responsible for avocado root rot, a disease primarily affecting the roots. Initial symptoms include dark brown, brittle, easily detached feeder roots (Gastañadui et al., 2021). Secondary symptoms manifest as leaf yellowing, progressive defoliation, and epinasty. This Oomycete is managed with fungicides such as fosetyl-Al and metalaxyl; however, prolonged use of these products reduces their efficacy as the pathogen develops resistance (de Andrade Lourenço et al., 2022; Salvatore et al., 2020). For these reasons, biological control is a fundamental tool in the integrated management against *P. cinnamomi*, because it can activate plant resistance mechanisms. Among the defense mechanisms of avocado against *P. cinnamomi*, the modification of the cell wall stands out, highlighting the biosynthesis of callose as a barrier to infection by the pathogen (Van den Berg et al., 2018). Likewise, among the early induced responses, the biosynthesis of salicylic acid (SA) and the positive regulation of the jasmonate to ethylene ratio (JA/ET) are of great importance against the biotrophic and necrotrophic action of the pathogen (Van den Berg et al., 2021). On the other hand, it has been shown that in resistant patterns the production of endochitinases and apoplastic proteases degrade extracellular proteins and limit the spread of *P. cinnamomi* (Engelbrecht and Van den Berg, 2013).

*Lasiodiplodia theobromae* is the causal agent of dieback disease, which severely impacts avocado crops at all stages of development, causing branch dieback, canker formation, and fruit peduncle necrosis (Jiménez-Ariza et al., 2023). In young plantations, infections often occur at the grafting site, leading to high plant mortality rates. The significance of this disease lies in the challenges associated with managing infections caused by this pathogen (Rodríguez-Gálvez et al., 2025). As an opportunistic pathogen, *L. theobromae* can persist in soil and pruning debris for extended periods, facilitating avocado plant reinfection (Ramírez-Gil and Osorio, 2021). Fungicides, the primary control measure, have limited efficacy, and the residues left on the fruit restrict export potential (Contreras et al., 2015).

Research has increasingly highlighted the importance of plant growth-promoting rhizobacteria (PGPR) as an alternative to chemical methods for crop disease control (Miyambo et al., 2022). Among the diverse bacteria with antifungal activity and growth-promoting properties, the genera *Pseudomonas* and *Bacillus* are particularly notable (Raaijmakers et al., 2002; De-Bashan et al., 2007).

*Pseudomonas* and *Bacillus* have been extensively studied as biological control agents. These rhizobacteria possess the unique ability to colonize the entire ecological niche of plant roots across all developmental stages, even in the presence of competing microorganisms (Antoun and Prévost, 2006). Communication between bacteria and plants occurs through chemotaxis, whereby plants release exudates that stimulate the active movement of flagellated bacteria, enabling colonization of the root epidermis (Kapulnik and Okon, 2002). Additionally, these rhizobacteria employ various mechanisms of action, including antibiotic synthesis, hydrolytic enzyme production, stimulation of systemic acquired resistance, and root biostimulation (Etebu and Osborn, 2012; Jena, 2012; Ramírez R. et al., 2015; Memenza et al., 2016; Weller, 2007).

For these reasons, this research aimed to determine the *in vitro* antagonistic activity of PGPR from the genera *Pseudomonas* and *Bacillus*, native to ‘Zutano’ avocado trees from Peru’s northern coast, against *Lasiodiplodia theobromae* and *Phytophthora cinnamomi*. Additionally, it sought to evaluate the efficacy of *Bacillus subtilis* application in mitigating the damage caused by *P. cinnamomi* and its beneficial effects on avocado plant growth.

## 2 Materials and methods

### 2.1 Counting and isolation of rhizobacteria from the genera *Pseudomonas* and *Bacillus*

Five rhizospheric soil samples associated with *Persea americana* variety Hass, grafted onto the Zutano rootstock, were collected at a 20 cm depth from avocado plantations in Virú, La Libertad, Peru, a region of significant agricultural productivity. The rhizospheric soil had the following physical-chemical characteristics: pH 6.57, EC 1.16 dS∙m^-1^, 0.1% CaCO₃, 0.57% organic matter, 26.7 mg∙kg^-1^ P, and 24 mg∙kg^-1^ K. From each sample, 10 g of soil was weighed, diluted, and agitated in 90 mL of peptonized water for 15 minutes at 15.71 rad∙s^-1^. Serial dilutions from 10^-1^ to 10^-7^ were prepared, and 0.1 mL of each dilution was plated on Petri dishes containing asparagine broth (Cetrimide) for *Pseudomonas* counts. The asparagine broth was composed of 1.4 g L^-1^ of potassium chloride, 10 g L^-1^ of potassium sulphate, 10 ml of glycerol, 0.3 g L^-1^ of cetrimide and 15 g L^-1^ of Agar, buffered to pH 7 ± 0.2. For bacillus counts we used Tryptone Glucose Extract (TGE) (Martínez, 2010). The recipe for TGE consisted of 5 g L^-1^ enzymatic digest of casein, 1 g L^-1^ dextrose and 15 g L^-1^ Agar, buffered to pH 7 ± 0.2.

For probable *Pseudomonas*, representative colonies displaying diverse morphological characteristics were selected from the range of microorganisms growing on cetrimide agar, and strains were isolated through successive sub culturing. *Pseudomonas* strains were characterized on cetrimide medium after 48 hours of incubation. For *Bacillus*, a representative selection of colonies with differing morphological traits was made from microorganisms grown on TGE medium, and the purification of potential *Bacillus* isolates was conducted on the same medium. One colony was selected from each isolate, streaked on nutrient agar to ensure the purity of each isolate, and stored at 4 °C for further testing.

### 2.2 Molecular identification of rhizobacteria

Molecular characterization of 25 bacterial isolates, grown overnight in nutrient broth at 37 °C, for 18 hours, was conducted. The nutrient broth consisted of 5 g of meat peptone, 3 g of meat extract, buffered to pH 7 ± 0.2. Genomic DNA from each isolate was extracted using the JetFlex™ Genomic DNA Purification Kit (Thermo Scientific), following the manufacturer’s protocol. Identification was achieved through 16S rRNA gene sequencing. PCR amplification, product purification and sequencing were conducted by Macrogen.

Gene sequence data were edited using CodonCode Aligner v. 8.02. Sequence alignment was conducted with Clustal W v. 2.0 (Larkin et al., 2007), and sequence similarity searches were performed using the Basic Local Alignment Search Tool (BLAST) (Altschul et al., 1990). The resulting sequences were compared with microbial sequences in the National Center for Biotechnology Information (NCBI) database.

### 2.3 *Phytophthora cinnamomi* and *Lasiodiplodia theobromae* inoculation

The *Phytophthora cinnamomi* isolate used in this study was provided by the Phytopathology Laboratory of the Faculty of Agronomy, National Agrarian University La Molina (UNALM). Mycelial propagation was initiated from the Petri dish containing the parent culture, making the necessary touches to obtain the pathogen. For this, 8 mm agar discs containing mycelium from the parent culture were placed on corn meal agar (CMA) (Mamani and Aragón, 2018). Zoospore production was carried out on disinfected carnation petals, immersed in 1% sodium hypochlorite for 60 seconds and then thoroughly rinsed in sterile water. The sterile petals were transferred to a 250 mL flask with 100 mL of sterile mineral water. Agar discs with *P. cinnamomi* were placed on the petals to facilitate pathogen colonization and inoculation of the pots. Inoculation of seedlings with *P. cinnamomi* was carried out by applying 30 ml of zoospore suspension at a concentration of 10^5^ zoospores ml^-1^, per pot (Rodríguez-Molina et al., 2003).

The *Lasiodiplodia theobromae* isolate used in this study was provided by the Phytopathology Laboratory at the Agronomy School of San Luis Gonzaga de Ica National University. The isolate was reactivated as described above and cultured on potato dextrose agar (PDA) (Contreras et al., 2015).

### 2.4 *In vitro* antagonistic activity of *Pseudomonas* and *Bacillus* against *Phytophthora cinnamomi* and *Lasiodiplodia theobromae*

The ability of the isolated bacteria to reduce mycelial growth of *Phytophthora cinnamomi* and *Lasiodiplodia theobromae* was evaluated with the help of dual culture assays (Moradi et al., 2018). For this, an 8 mm agar plug of a 4-day old *P. cinnamomi* isolate was placed in the center of a Petri dish containing CMA. A 20 µL of inoculum from bacterial isolates, cultured for 48 hours in nutrient broth at a population density of 10^8^ CFU∙mL^-1^, was streaked at three equidistant points around the fungal plug, each point serving as a replicate. The same procedure was followed for *L. theobromae*, grown on PDA plates. Petri dishes were incubated at 25 °C in the dark and growth inhibition percentages (Equation 1) were measured for both isolates after 72 hours (Orberá et al., 2014).

(1) $$\%Inhibition=\frac{\left(R-r\right)}{R}\;x\;100$$

In plates containing three bacterial streaks, the maximum fungal colony radius (R) was measured in the direction showing unrestricted fungal growth, whereas the inhibited fungal colony radius (r) was measured from the center of the fungal plug toward the bacterial streak where fungal growth was restricted.

r: fungal colony radius measured toward the bacterial streak.

R: is the maximum pathogen colony radius.

### 2.5 Antagonistic activity of *Bacillus subtilis* on *Phytophthora cinnamomi*

The greenhouse experiment was conducted in a completely randomized design (DCA), with three treatments and five replicate plants. The first treatment was the control without *Phytophthora cinnamomi* inoculation. The second treatment was the treatment with *P. cinnamomi* inoculation without *Bacillus subtilis*. The third treatment was the co-inoculation treatment of *P. cinnamomi* and *B. subtilis*. Seedling infection with *P. cinnamomi* was achieved by applying 30 mL of zoospore suspension at a concentration of 10⁵ zoospores∙mL^-1^ per pot (Rodríguez-Molina et al., 2003). Avocado seeds of the ‘Zutano’ variety, weighing approximately 50 g and donated by Avo Hass Peru, were planted in 2 L plastic pots. Premix^®^ No. 8 substrate was used as the growth medium (pH: 5.5, EC: 0.75 dS∙m^-1^, P: 25 mg∙kg^-1^, and K: 100 mg∙kg^-1^). Before sowing, the seeds were disinfected in 10% sodium hypochlorite solution for 10 minutes and soaked for 24 hours in bacterial broth prepared from *B. subtilis* isolate Bac F. We used *B. subtilis* isolate Bac F because it was the greatest inhibitor against the pathogen on *in vitro* essays.

This bacterial culture was incubated for 48 hours in nutrient broth at a concentration of 10^8^ CFU∙mL^-1^. The seeds and broth were contained in sterile bags, with 50 mL of broth allocated per seed.

Once germinated, the seeds were cultivated in a greenhouse for three weeks at an average temperature of 20°C and a relative humidity of 60% until seedlings developed. Subsequently, 50 mL of the bacterial isolate Bac F of *Bacillus subtilis*, cultured in nutrient broth at 10^8^ CFU∙mL^-1^, was applied. One week later, infection of seedlings with *Phytophthora cinnamomi* was carried out by applying 30 ml of zoospore solution at a concentration of 10^5^ zoospores ml^-1^, maintaining constant soil moisture throughout the experiment.

Three weeks after inoculation, successful infection by *Phytophthora cinnamomi* was confirmed by staining infected root tissues with trypan blue following the method of Del Castillo-González et al. (2024). The presence of characteristic pathogen structures within root tissues was used to verify pathogen colonization in inoculated plants. Root growth parameters associated with pathogen damage, including weight, length, and moisture content, were evaluated following the methodology of Gómez and Apaza (2015). Additionally, the number of leaves and aerial biomass were recorded. The severity of *Phytophthora cinnamomi* infection in avocado seedlings pre-inoculated with the *Bacillus subtilis* isolate Bac F was determined using the calibrated visual scale for pot studies, proposed by Ramírez-Gil and Morales-Osorio (2020a) (**Table 1**), based on external symptoms.

**Table 1:** Description of the calibrated disease developmental scale visual for pot studies for *Phytophthora cinnamomi* in avocado cv. Hass (Ramírez-Gil and Morales-Osorio, 2020a).

Scale value	Appearance of the shoot and disease symptoms	Appearance of roots
0	Healthy plants with abundant foliage of dark green color and foliar buds in active growth. No disease symptoms	>90% of viable rootlets
1	Visible disease symptoms. Mild leaf yellowing and lack of active growing buds lead to stunted growth	Diseased rootlets between 10 and 15%
2	Pronounced leaf yellowing and stunted growth	Diseased rootlets between 15.1 and 25%
3	Generalized leaf yellowing, wilting, and mild defoliation<35%	Diseased rootlets between 25.1 and 50%

### 2.6 Statistical analysis

Statistical analyses were conducted using IBM’s Statistical Package for Social Sciences (SPSS), version 24. Prior to ANOVA, data normality and homogeneity of variances were verified using the Shapiro–Wilk and Levene’s tests, respectively. Data from each experiment were then subjected to analysis of variance (ANOVA), and treatment means were compared using Duncan’s multiple range test at a 95% confidence level. An alpha error probability of less than 5% was considered statistically significant.

## 3 Results

### 3.1 Molecular identification of *Bacillus* and *Pseudomonas* isolates

A total of 25 isolates were obtained, including 14 probable *Pseudomonas* isolates cultured on Cetrimide Agar and 11 probable *Bacillus* isolates grown on TGE and (**Table 2**). Amplification of the 16S rDNA gene in the identified bacterial isolates produced bands ranging from 1197 to 1863 bp. The similarity between the nucleotide sequences obtained and the sequences from the Gen Bank database by BLAST analysis showed that the isolates belong to the *Pseudomonaceae* and *Bacillaceae* families, and are related to the *Pseudomonas*, *Bacillus*, and *Lysinibacillus* genera, respectively (**Table 2**).

**Table 2:** Rhizobacteria molecular identification isolated from *Persea americana*, based on 16S rRNA gene sequencing.

Isolation	Identity (BLAST NCBI)	GENEBANK access number	Similarity (%)
P1	*Pseudomonas putida*	MT982622	99
P2	*Pseudomonas putida*	MT982623	99
P3	*Pseudomonas putida*	MT982624	99
P4	*Pseudomonas putida*	MT982625	99
P6	*Pseudomonas putida*	MT982626	99
P7	*Pseudomonas putida*	MT982627	99
P10	*Pseudomonas putida*	MT982628	99
P11	*Pseudomonas* sp.	MT982629	99
P12	*Pseudomonas putida*	MT982630	99
P14	*Pseudomonas putida*	MT982631	99
BAC A	*Lysinibacillus macroides*	MT982632	99
BAC B	*Lysinibacillus xylanilyticus*	MT982633	99
BAC C	*Lysinibacillus fusiformis*	MT982634	99
BAC D	*Lysinibacillus fusiformis*	MT982635	99
BAC E	*Lysinibacillus xylanilyticus*	MT982636	99
BAC F	*Bacillus subtilis*	MT982637	99
BAC G	*Lysinibacillus xylanilyticus*	MT982638	99
BAC H	*Bacillus* sp.	MT982639	99
BAC I	*Bacillus* sp.	MT982640	99
BAC J	*Lysinibacillus fusiformis*	MT982641	99
BAC K	*Lysinibacillus* sp.	MT982642	98
BAC L	*Pseudomonas* sp.	MT982643	99
BAC M	*Pseudomonas plecoglossicida*	MT982644	99
BAC N	*Pseudomonas* sp.	MT982645	99
BAC O	*Pseudomonas* sp.	MT982646	99

### 3.2 *In vitro* antagonistic activity of *Pseudomonas* and *Bacillus* on *Phytophthora cinnamomi*

**Figure 1:**
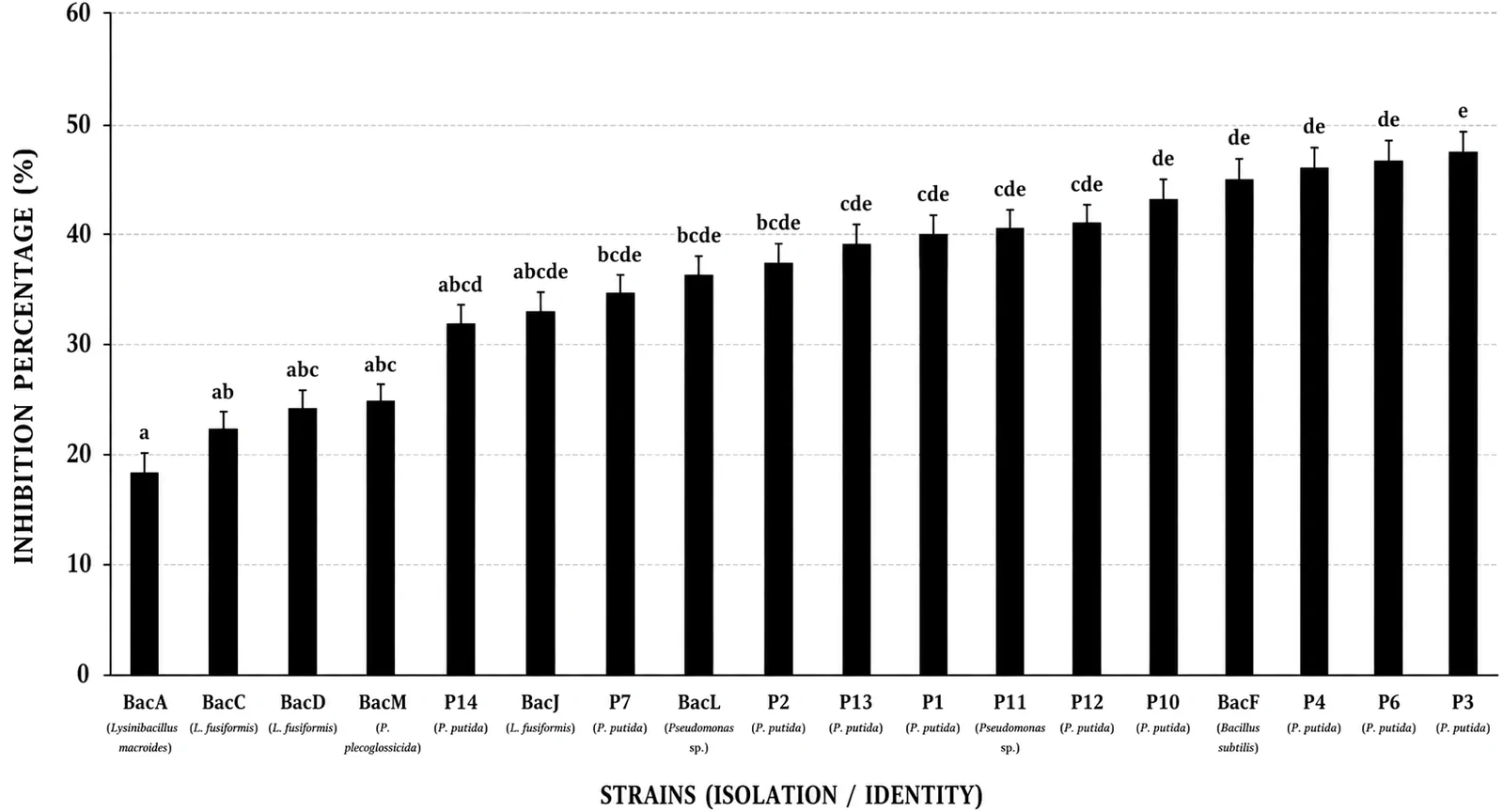
*In vitro* inhibition percentage of *Pseudomonas* and *Bacillus* isolated from the avocado rhizosphere against *Phytophthora cinnamomi* (p< 0.05).

**Figure 2:**
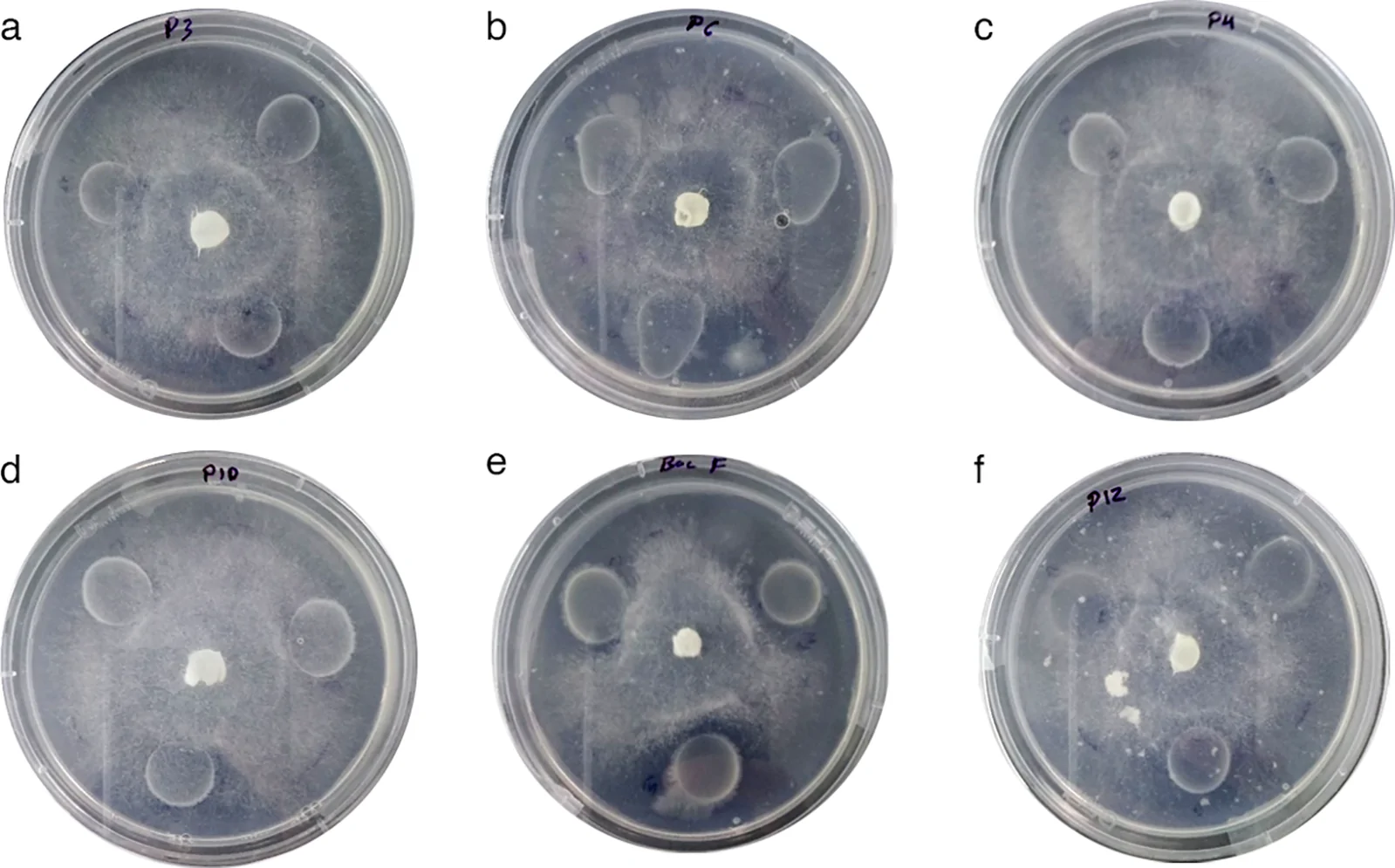
Bacterial isolates from the avocado rhizosphere showed the largest inhibition halos against *Phytophthora cinnamomi* four days post-inoculation. **(a)** *Pseudomonas putida* (P3), **(b)** *P. putida* (P6), **(c)** *P. putida* (P4), **(d)** *P. putida* (P10), **(e)** *Bacillus subtilis* (Bac F), and **(f)** *P. putida* (P12) (p< 0.05).

**Figure 3:**
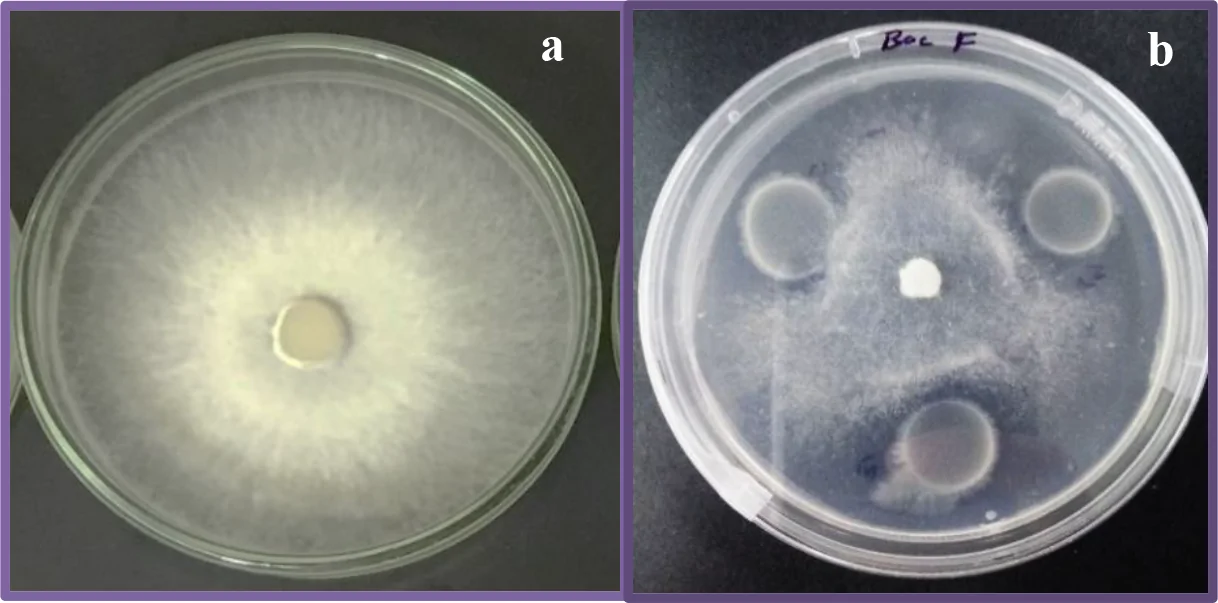
**(a)** Colonization of *Phytophthora cinnamomi* in the control treatment without inoculation and **(b)** the inhibition halos produced by the rhizospheric bacterium *Bacillus subtilis* (Bac F) on *P. cinnamomi* seven days post-inoculation.

The 25 isolates (**Table 2**) were tested for antagonistic activity against *Phytophthora cinnamomi* using dual plate culture, and only 18 isolates displaying inhibitory effects. **Figure 1** shows the inhibition percentage achieved by each bacterial isolate against the pathogen. Among these, *Pseudomonas* and *Bacillus* isolates demonstrated the highest inhibition halo four days after confrontation (**Figure 2**), with notable activity from *Pseudomonas putida* isolates P3, P6, P4, P10 and *Bacillus subtilis* isolate Bac F. However, *B. subtilis* isolate Bac F was the only one that maintained high inhibition capacity, achieving 44% inhibition at 7 days of evaluation (**Figure 3**).

### 3.3 *In vitro* antagonistic activity of *Pseudomonas* and *Bacillus* on *Lasiodiplodia theobromae*

**Figure 4:**
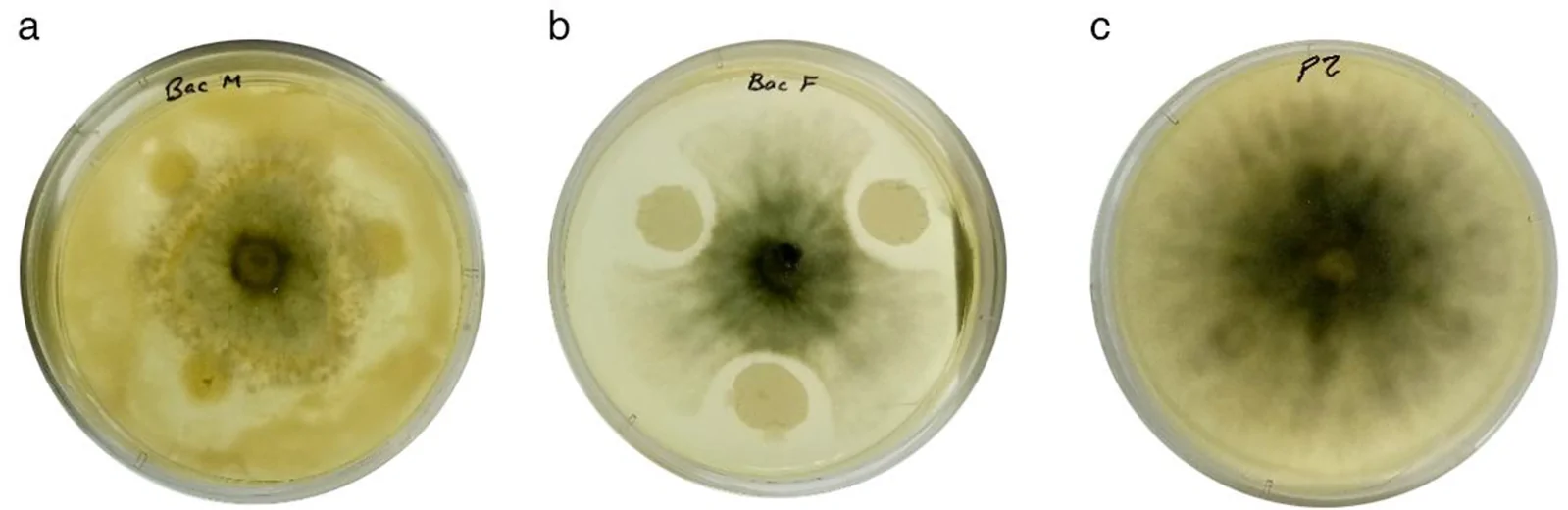
Inhibition halos produced by avocado rhizobacteria against *Lasiodiplodia theobromae* 3 days after inoculation, including **(a)** *Pseudomonas plecoglossicida* (Bac M), **(b)** *Bacillus subtilis* (Bac F), and **(c)** the control treatment without inoculation.

**Figure 5:**
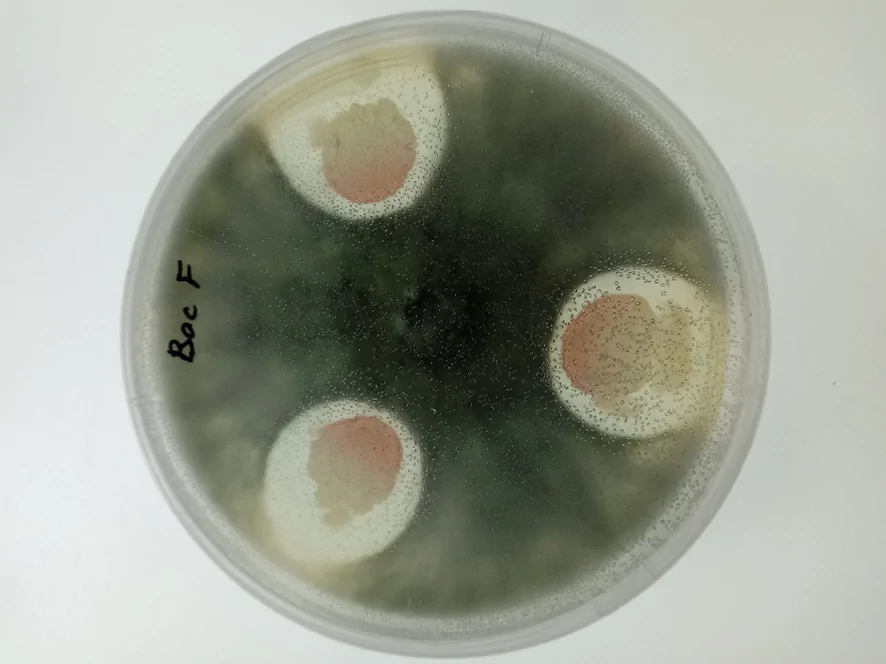
Inhibition halos produced by the rhizospheric bacterium *Bacillus subtilis* (Bac F), isolated from avocado, against *Lasiodiplodia theobromae* 7 days after inoculation.

The 25 isolates were evaluated for antagonistic activity against *Lasiodiplodia theobromae* in dual plate culture, of which only two exhibited such activity (**Figure 4**). *Pseudomonas plecoglossicida* isolate Bac M inhibited the mycelial growth of *L. theobromae* during four days after confrontation, but its rapid expansion rate allowed it to fill the plate before Bac M could establish itself. For that, this effect was temporary, as *L. theobromae* invaded *P. plecoglossicida* (Bac M) after 7 days. In contrast, *Bacillus subtilis* isolate Bac F also inhibited the mycelial growth of *L. theobromae* after 4 days, and this effect persisted through the 7-day evaluation period (**Figure 5**), resulting in an inhibition percentage of 33.33%.

### 3.4 *In vivo* antagonistic activity of *Bacillus subtilis* isolate Bac F on *Phytophthora cinnamomi*

**Figure 6:**
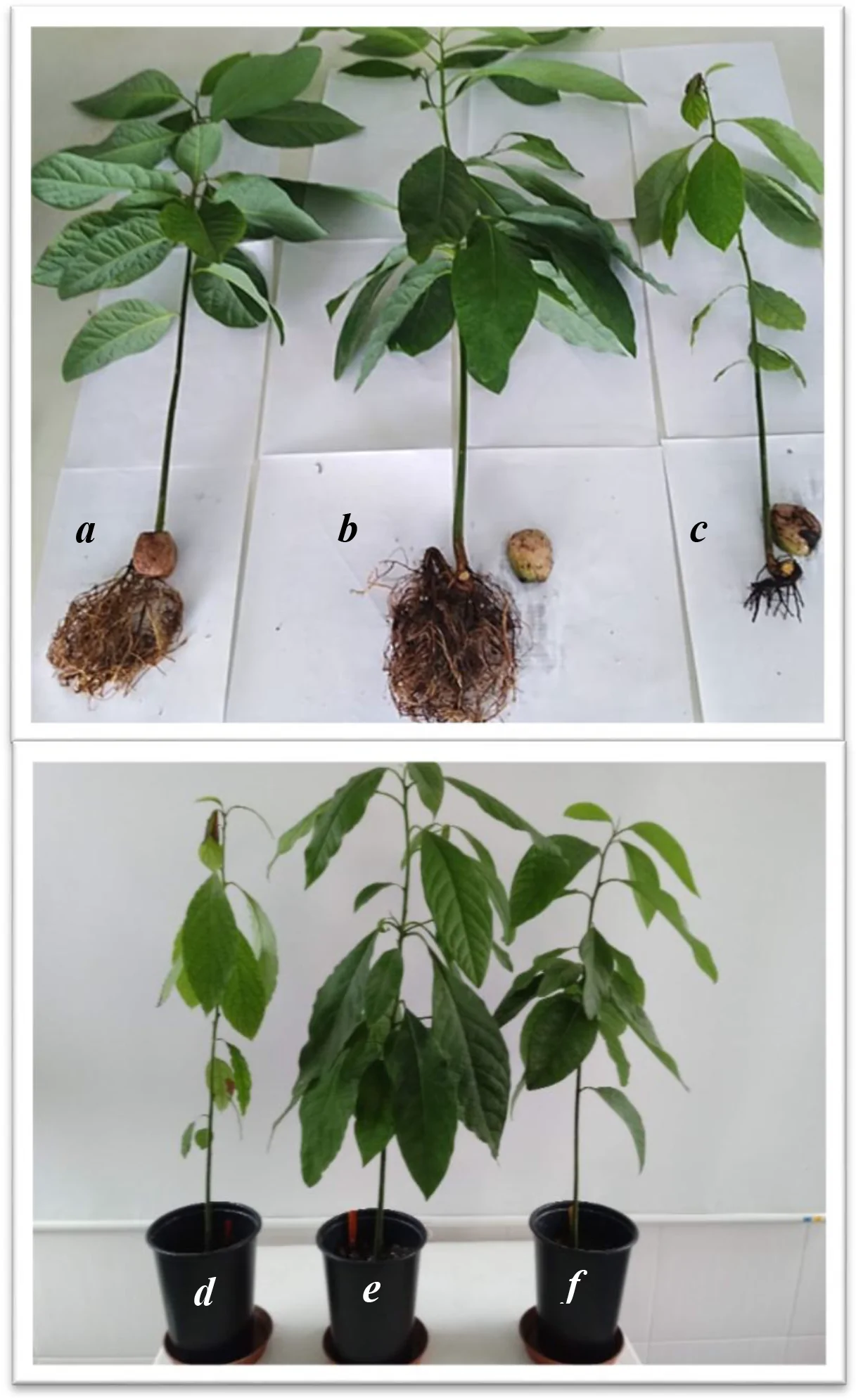
Antagonistic effect of *Bacillus subtilis* against *Phytophthora cinnamomi* in avocado plants. Root system evaluation: **(a)** Plant inoculated with *P. cinnamomi* and *B. subtilis*; **(b)** Control plant without inoculation; **(c)** Plant inoculated with *P. cinnamomi* only. Vegetative growth evaluation: **(d)** Plant inoculated with *P. cinnamomi* only; **(e)** Control plant without inoculation and **(f)** Plant inoculated with *P. cinnamomi* and *B. subtilis*.

The results indicated that immersing avocado seeds for 24 hours in 50 mL of *Bacillus subtilis* (Bac F) bacterial broth (10^8^ CFU∙mL^-1^), combined with an application of the same broth at a dosage of 50 mL per plant 3 weeks after germination, provided significant preventative control of *Phytophthora cinnamomi* (**Table 3**). According to the visual disease severity scale established by Ramírez-Gil and Morales-Osorio (2020a), seedlings inoculated with *B. subtilis* were classified as scale 0, corresponding to healthy plants with more than 90% viable rootlets, no visual symptoms of chlorosis, and less than 10% root damage. In addition, plants inoculated only with *P. cinnamomi* exhibited severe root deterioration, with visual observations indicating that more than 70% of the root system was affected by rot. This observation was recorded as a complementary assessment of root damage and was not used to modify the disease severity categories defined by the visual scale of Ramírez-Gil and Morales-Osorio (2020a). Furthermore, this treatment mitigated the adverse effects of *P. cinnamomi* on both root and vegetative growth in avocado plants, as illustrated in **Figure 6**. **Table 4** presents that the co-inoculation treatment of *P. cinnamomi* with *B. subtilis* achieved an increase of 116.58% and 44.59% in root dry weight and root length, respectively, compared to the inoculation treatment of *P. cinnamomi* without *B. subtilis*. Additionally, the inoculation of *B. subtilis* enhanced vegetative growth, leading to a 19.26% increase in the number of leaves and a 93.26% increase in aerial dry weight, compared to the inoculation without *B. subtilis*.

**Table 3:** Scale used to evaluate root rot severity in avocado plants caused by *Phytophthora cinnamomi* under greenhouse conditions, based on disease symptoms and root health across different treatments.

Treatment	Scale value	Outbreak appearance and disease symptoms	Root appearance
Control (without P. cinnamomi)	0	Healthy plants with abundant dark green foliage and actively growing leaf buds. No symptoms of illness	> 90% of viable rootlets.
P. cinnamomi	3	Generalized chlorosis, wilting and defoliation.	Rotten roots > 70%.
P. cinnamomi + B. subtilis	0	Healthy plants with abundant dark green foliage and actively growing leaf buds. No symptoms of illness	> 90% of viable rootlets.

**Table 4:** Effect of the *Bacillus subtilis* bacterial isolate Bac F on root growth parameters associated with damage caused by the pathogen *Phytophthora cinnamomi* in avocado plants.

Treatment	Root fresh weight (g)	Root dry weight (g)	Root relative humidity (g)	Root length (cm)	Root length/root dry weight	Number of leaves	Aerial fresh weight (g)	Aerial dry weight (g)
Control (without *P. cinnamomi*)	21.9 c	4.60 b	17.3 b	27.2 b	5.97 ab	23.33 b	60.67 b	29.71 b
*P. cinnamomi*	10.41 a	2.11 a	8.29 a	14.8 a	7.63 b	19.00 a	39.71 a	13.64 a
*P. cinnamomi + B. subtilis*	15.08 b	4.57 b	10.5 a	21.4 ab	5.23 a	22.66 b	61.37 b	26.36 b

Identical letters next to the values of each evaluated variable indicate no significant statistical difference (Duncan, P = 0.05).

**Figure 7:**
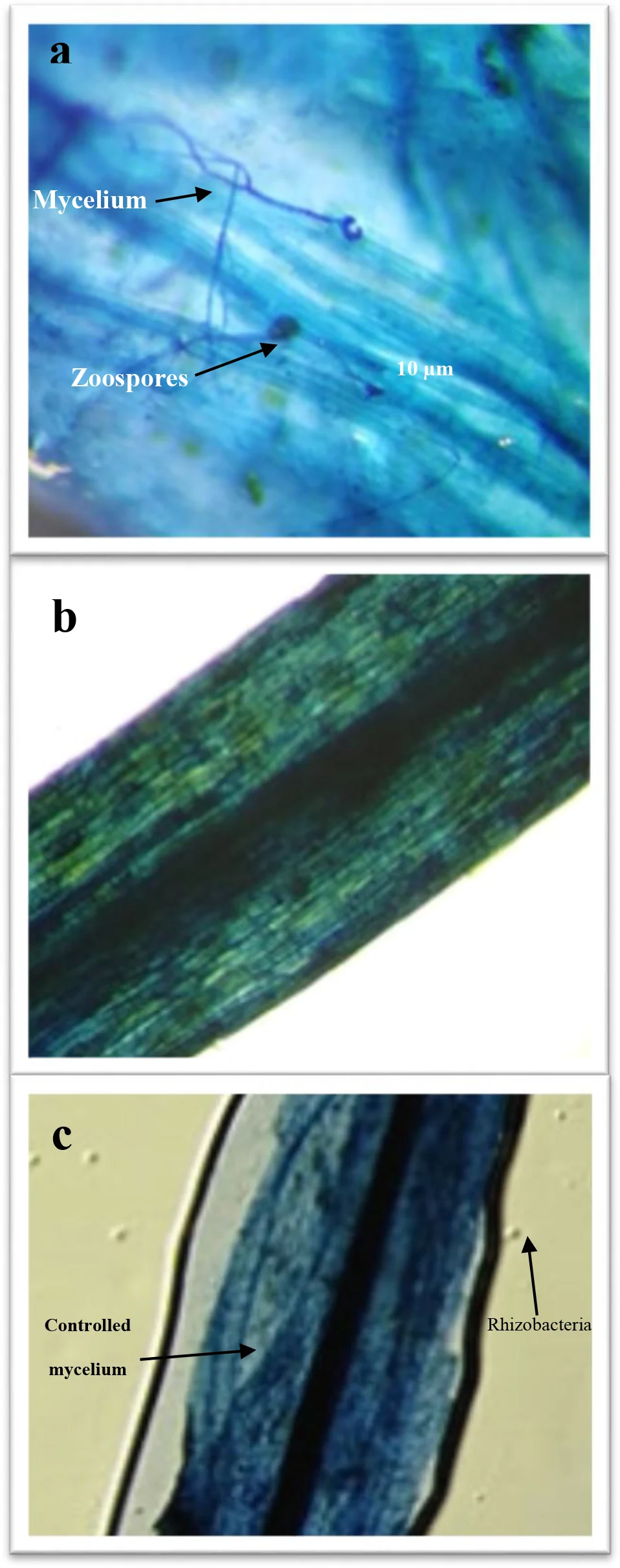
Confirmation of *Phytophthora cinnamomi* infection by trypan blue staining of avocado roots. **(a)** Root tissues from plants inoculated with *P. cinnamomi* showing characteristic pathogen structures (400×); **(b)** non-inoculated control roots (400×); **(c)** roots from plants inoculated with *P. cinnamomi* and treated with *Bacillus subtilis* (1000×).

Successful infection by *Phytophthora cinnamomi* was confirmed by trypan blue staining of root tissues (**Figure 7**), which revealed characteristic pathogen structures in inoculated plants. No pathogen structures were observed in the non-inoculated control plants. It is important to note that *P. cinnamomi* was inoculated under axenic conditions, allowing for the precise observation of *Bacillus subtilis* activity against the pathogen. **Figure 7** illustrates *B. subtilis* inhibition of *P. cinnamomi* mycelial development.

## 4 Discussion

The results of this study demonstrated the antagonistic effects of *Bacillus* and *Pseudomonas*, isolated from the rhizosphere of *Persea americana* in productive fields on the northern coast of Peru, against *Phytophthora cinnamomi* and *Lasiodiplodia theobromae* on *in vitro* conditions. Also, we present that *Bacillus subtilis* demonstrated substantial growth-promoting capacity by inhibiting *P. cinnamomi* development and promoting robust root and aerial growth in avocado plants.

*Bacillus subtilis* showed one of the highest inhibition of *Phytophthora cinnamomi*, with a 44% reduction observed 7 days after *in vitro* treatment. In a related study, *Bacillus acidiceler*, isolated from the rhizosphere of avocado trees exhibiting decline symptoms, inhibited *P. cinnamomi* by 76% through the production of volatile compounds, including 2,3,4-trimethylpyrazine, 6,10-dimethyl-5,9-undecadiene-2-one, and 3-amino-1,3-oxazolidine-2-one (Méndez-Bravo et al., 2018). Ramírez-Gil and Morales-Osorio, (2020b) reported similar findings, where Bacillus sp. achieved 45% *in vitro* inhibition of P. cinnamomi, which is comparable to the 44% inhibition observed in the present study. The beneficial effects of *Bacillus* have also been observed in other crops, such as potatoes, specifically against *Rhizoctonia solani* (Calvo et al., 2010) and in controlling other phytopathogenic fungi (Ongena et al., 2005; Ramírez‐Gil et al., 2017). Integrated management programs incorporating *Bacillus*, along with bio rational strategies, chemicals, and an energy source, have been shown to reduce the disease progression curve and inoculum load of *P. cinnamomi* in avocado crops by up to 67.2% and 44.3%, respectively, in greenhouse conditions, and by 74.8% and 58.5% in productive field conditions (Ramírez-Gil and Morales-Osorio, 2020b). Notably, *Phytophthora* species are among the most aggressive oomycetes in agriculture, requiring both chemical and biological inhibitors for short-term control, and it is necessary to include inoculation of *B. subtilis* into management programs (de Andrade Lourenço et al., 2022).

*Pseudomonas putida* demonstrated the highest inhibition of *Phytophthora cinnamomi* until the fourth day of incubation, reaching 47%. However, this inhibition was not sustained until the seventh day of evaluation. Mamani and Aragón (2018) reported different results using isolates of *Pseudomonas* sp. from the rhizosphere of avocado in Casma, where inhibition ranged from 28.3% to 47% and lasted longer across eight selected isolates. The inhibition mechanism involved the disintegration of the cytoplasmic contents of *P. cinnamomi* mycelial cells, likely due to the release of hydrolytic enzymes like glucanases and lipases, which cause extensive lysis of *P. cinnamomi* mycelium *in vitro* (Dubey et al., 2017). Another potential contributor to the antagonistic effect of *Pseudomonas* is the release of antibiotic compounds such as pyolterin, pyrrolnitrin, and phenazine derivatives (Shahid et al., 2021). *Pseudomonas aurantiaca* has been found to inhibit mycelial growth of *P. cinnamomi* by producing phenazine-derived compounds, 2-undecanone, and ammonia (Zhang et al., 2022). The transient effect of *P. putida* on *P. cinnamomi* inhibition may be due to the need for culture medium optimization, specifically by selecting a more effective carbon source that enhances inhibitory substance production (Kong et al., 2023). It was found that *Pseudomonas aurantiaca* inoculated on a medium with 20.59 g∙L^-1^ glucose and 18.56 g∙L^-1^ yeast extract powder increased inhibition of *P. cinnamomi* to 76.1% after 7 days of incubation (Kong et al., 2023).

Regarding the inhibition of *Lasiodiplodia theobromae*, *Pseudomonas plecoglossicida* suppressed mycelial growth for up to 4 days, though this effect was not sustained. In contrast, *Bacillus subtilis* exhibited greater efficacy, achieving an inhibition rate of 33.33% for up to 7 days. *Bacillus amyloliquefaciens*, a closely related species to *B. subtilis*, demonstrated even higher efficacy, with 58.89% inhibition against *L. theobromae* out of 890 evaluated isolates (Chukeatirote et al., 2018). *B. subtilis* reduces plant susceptibility to fungal pathogens by enhancing host resistance through differential gene expression (Ongena et al., 2005). The *Pseudomonas* genus is notable for producing a wide array of bioactive secondary metabolites, including cyclic lipopeptides and biosurfactants with antimicrobial properties against various plant pathogens (El-Sayed et al., 2018). Lipopeptides (LPs), which serve as protective agents against *Pseudomonas* and *Bacillus* predators, provide a competitive advantage over other microorganisms and can also elicit defense responses in plants by stimulating phytoalexin production (Dubey et al., 2017).

The results of this study indicate that inoculating *Bacillus subtilis* in avocado plants during the greenhouse phase effectively prevented or reduced root rot caused by *Phytophthora cinnamomi*, successfully controlling symptoms associated with the pathogen (**Tables 3**, **4**; **Figure 6**). Both *Bacillus* and *Pseudomonas* have been extensively documented for promoting plant growth through mechanisms such as siderophore and phytohormone production, phosphate solubilization, nitrogen fixation, and antimicrobial activities (Gupta et al., 2015; Sarbadhikary and Mandal, 2017; Singh et al., 2015; Zamioudis et al., 2013). Plant growth-promoting rhizobacteria (PGPRs) primarily enhance root branching through the action of N-acyl-L-homoserine lactones (AHLs), cyclodipeptides (CPDs), and volatile organic compounds (Ortiz-Castro et al., 2017). Mamani and Aragón (2018) found that *P. cinnamomi* can cause up to 77.47% root damage, which can be reduced to 44.84–69.04% depending on the *Pseudomonas* sp. isolate. These authors also noted that certain *Pseudomonas* strains stimulate growth under *P. cinnamomi*-induced biotic stress, enhancing root length, root dry weight, and leaf biomass. This technique offers potential as a more environmentally friendly alternative to commercial agrochemicals for disease control and the development of avocado seedlings in greenhouses, improving success rates when transplanted to the final field.

Future studies should validate the efficacy of *B. subtilis* under larger greenhouse experiments and field conditions to confirm the consistency and applicability of these findings. In addition, quantitative methods for assessing root viability should be incorporated to complement the visual disease severity scale used in this study. Further research should also investigate the molecular and biochemical mechanisms underlying the antagonistic activity of *B. subtilis* to support its long-term application in integrated disease management of avocado.

## 5 Conclusions

*Bacillus subtilis* (Bac F) was the bacterial isolate with the most prolonged antagonistic activity against *Phytophthora cinnamomi* and *Lasiodiplodia theobromae*, reaching inhibition percentages of 44% and 33.33%, respectively. Furthermore, inoculation with *B. subtilis* in avocado plants infected with *P. cinnamomi* resulted in 90% root viability and increased root dry weight and root length by 116.58% and 44.59%, respectively. These results demonstrate the potential of *B. subtilis* as a biological control agent against *P. cinnamomi* in avocado, while *Pseudomonas putida* showed promising *in vitro* antagonistic activity that warrants further evaluation under greenhouse and field conditions.

## Data Availability

The raw data supporting the conclusions of this article will be made available by the authors, without undue reservation.

## References

[B1] Altschul S. F. Gish W. Miller W. Myers E. W. Lipman D. J. (1990). Basic local alignment search tool. J. Mol. Biol. 215, 403–410. doi: 10.1016/S0022-2836(05)80360-2 2231712

[B2] Antoun H. Prévost D. (2006). “ Ecology of plant growth promoting rhizobacteria,” in Pgpr: Biocontrol and Biofertilization. Ed. Siddiqui Z. A. ( Springer, Dordrecht), 1–38.

[B3] Barrientos-Priego A. F. Muñoz-Pérez R. Reyes-Alemán J. Borys M. W. Martínez-Damián M. T. (2007). “ Taxonomía, cultivares y portainjertos. el aguacate y su manejo integrado,” in Segunda Edición. Mundi Prensa. Eds. Téliz D. Mora A. (México, DF: Mundi-Prensa México, S.A. de C.V.), 31–62.

[B4] Calvo P. Ormeño-Orrillo E. Martínez-Romero E. Zúñiga D. (2010). Characterization of Bacillus isolates of potato rhizosphere from andean soils of Peru and their potential PGPR characteristics. Braz. J. Microbiol. 41, 899–906. doi: 10.1016/0921-5093(91)90903-z 24031569 PMC3769774

[B5] Chukeatirote E. Phueaouan T. Piwkam A. (2018). Screening of rhizosphere soil bacteria for biocontrol of Lasiodiplodia theobromae. Agric. Nat. Resour. 52, 325–329. doi: 10.1016/j.anres.2018.10.009 38826717

[B6] Contreras S. S. Espejo M. R. Ruiz J. C. (2015). Actividad antifúngica del extracto etanólico de las hojas de Schinus molle sobre el crecimiento de Lasiodiplodia theobromae en condiciones de laboratorio. Rev. Investigación Científica REBIOL 35, 47–52.

[B7] de Andrade Lourenço D. Branco I. Choupina A. (2022). A systematic review about biological control of phytopathogenic Phytophthora cinnamomi. Mol. Biol. Rep. 49, 9947–9962. doi: 10.1007/s11033-022-07547-2 35585380

[B8] De-Bashan L. E. Holguin G. Glick B. R. Bashan Y. (2007). “ Bacterias promotoras de crecimiento en plantas para propósitos agrícolas y ambientales,” in Microbiología Agrícola. Hongos, Bacterias, Micro Y Macrofauna, Control Biológico, Planta-Microorganismo ( Editorial Trillas, México), 170–224.

[B9] Del Castillo-González L. Soudani S. De La Cruz-Gómez N. Manzanera J. A. Berrocal-Lobo M. (2024). An improved method to study Phytophthora cinnamomi Rands zoospores interactions with host. BMC Plant Biol. 24, 508. doi: 10.1186/s12870-024-05205-2 38844843 PMC11154991

[B10] Dubey R. K. Vishal Tripathi V. T. Edrisi S. A. Mansi Bakshi M. B. Dubey P. K. Ajeet Singh A. S. . (2017). “ Role of plant growth-promoting microorganisms in sustainable agriculture and environmental remediation,” in Advances in PGPR Research ( CABI, Wallingford UK), 75–125.

[B11] El-Sayed A. S. Akbar A. Iqrar I. Ali R. Norman D. Brennan M. . (2018). A glucanolytic Pseudomonas sp. associated with Smilax bona-nox L. displays strong activity against Phytophthora parasitica. Investigación microbiológica 207, 140–152. doi: 10.1016/j.micres.2017.11.018 29458848

[B12] Engelbrecht J. Van den Berg N. (2013). Expression of defence-related genes against Phytophthora cinnamomi in five avocado rootstocks. S. Afr. J. Sci. 109, 1–8. doi: 10.1590/sajs.2013/20120058

[B13] Etebu E. Osborn A. M. (2012). A review of indicators of healthy agricultural soils with pea footrot disease suppression potentials. Sustain. Agric. Res. 1, 235–250. doi: 10.5539/sar.v1n2p235

[B14] FAO (2023). FAOSTAT: Production of Avocados (Rome, Italy: Food and Agriculture Organization of the United Nations). Available online at: https://www.fao.org/faostat/en/#data/QCL (Accessed June 20, 2025).

[B15] Gastañadui P. Moreno R. Quiroz-Delgado P. E. Apaza-Tapia W. E. (2021). Control of avocado root rot caused by Phytophthora cinnamomi with different Trichoderma strains at Chavimochic Irrigation Project. Peruvian J. Agron. 5, 78–86. doi: 10.21704/pja.v5i3.1846

[B16] Gómez J. Apaza W. (2015). Reacción a la pudrición radicular causada por Phytophthora cinnamomi. Rands en dos razas y dos cultivares de palto, Persea americana. Miller (Lima: Universidad Nacional Agraria La Molina. Facultad de Agronomía).

[B17] Gupta G. Parihar S. S. Ahirwar N. K. Snehi S. K. Singh V. (2015). Plant growth promoting rhizobacteria (PGPR): Current and future prospects for development of sustainable agriculture. J. Microbial Biochem. Technol. 7, 96–102. doi: 10.4172/1948-5948.1000188

[B18] Jena N. (2012). Pseudomonas and Trichoderma: The most effective bio-control agents against damping off pathogens of vegetable crops. Asian J. Microbiology Biotechnol. Environ. Sci. 14, 295–298.

[B19] Jiménez-Ariza N. V. Soto-Heredia J. M. Aragón-Caballero L. M. (2023). Posible inducción de resistencia sistémica a Lasiodiplodia theobromae en aguacate (Persea americana Mill.) en condiciones semicontroladas en La Molina. Peruvian J. Agron. 7, 132–143. doi: 10.21704/pja.v7i2.2053

[B20] Kapulnik Y. Okon Y. (2002). “ Plant growth promotion by rhizosphere bacteria,” in Plant Roots (New York, NY: CRC Press), 1344–1368.

[B21] Kong W. L. Zhang Y. Wu X. Q. (2023). Optimization of Pseudomonas aurantiaca ST-TJ4 Fermentation Medium and Its Control Effect on Phytophthora cinnamomi. Fermentation 10, 21. doi: 10.21203/rs.3.rs-9242643/v1 42406807

[B22] Larkin M. A. Blackshields G. Brown N. P. Chenna R. McGettigan P. A. McWilliam H. . (2007). Clustal W and Clustal X version 2.0. Bioinformatics 23, 2947–2948. doi: 10.1093/bioinformatics/btm404 17846036

[B23] Mamani J. Aragón L. (2018). Pseudomonas of the rhizosphere of avocado (Persea americana Mill.) with biocontrol activity of Phytophthora cinnamomi Rands isolated in the central coast of Peru. Peruvian J. Agron. 2, 35–43. doi: 10.21704/pja.v2i3.1231

[B24] Martínez C. (2010). Diversidad y selección de Pseudomonas fluorescentes provenientes de la rizósfera y suelo como promotores de crecimiento y desarrollo de plantas de papa (Lima, Perú: UNALM), 32–49.

[B25] Martínez F. E. Deantonio L. Y. Aguilera E. Araujo G. Ortiz L. Rojas E. O. . (2015). “ Aptitud agroclimática e identificación de nichos productivos de bajo riesgo a deficiencias hídricas para aguacate en El Tambo, Colombia,” in Actas Viii Congreso Mundial De La Palta. Manejo De Técnicas Y De Cultivo, Lima, Peru: VIII World Avocado Congress Organizing Committee 342–348.

[B26] Memenza M. Mostacero E. Camarena F. Zúñiga D. (2016). “ Disease control and plant growth promotion (PGP) of selected bacterial strains in Phaseolus vulgaris,” in Biological Nitrogen Fixation and Beneficial Plant-Microbe Interaction (Cham: Springer International Publishing), 237–245.

[B27] Méndez-Bravo A. Cortazar-Murillo E. M. Guevara-Avendaño E. Ceballos-Luna O. Rodríguez-Haas B. Kiel-Martínez A. L. . (2018). Plant growth-promoting rhizobacteria associated with avocado display antagonistic activity against Phytophthora cinnamomi through volatile emissions. PloS One 13, e0194665. doi: 10.1371/journal.pone.0194665 29558512 PMC5860777

[B28] Miyambo T. M. Backer R. Engelbrecht J. Joubert F. van der Merwe N. A. Van den Berg N. (2022). The identification and characterization of endopolygalacturonases in a South African isolate of Phytophthora cinnamomi. Microorganisms 10, 1061. doi: 10.3390/microorganisms10051061 35630501 PMC9146145

[B29] Moradi M. Nejad F. J. Bonjar G. H. S. Fani S. R. Mimand B. M. Probst C. . (2018). Efficacy of Bacillus subtilis native strains for biocontrol of Phytophthora crown and root rot of pistachio in Iran. Trop. Plant Pathol. 43, 306–313. doi: 10.61838/kman.pwj.4.2.2

[B30] Ongena M. Duby F. Jourdan E. Beaudry T. Jadin V. Dommes J. . (2005). Bacillus subtilis M4 decreases plant susceptibility towards fungal pathogens by increasing host resistance associated with differential gene expression. Appl. Microbiol. Biotechnol. 67, 692–698. doi: 10.1007/s00253-004-1741-0 15578181

[B31] Orberá T. Serrat M. Ortega E. (2014). Potencialidades de la cepa SR/B-16 de Bacillus subtilis para el control de enfermedades causadas por hongos en cultivos de interés agrícola. Biotecnología Aplicada 31, 7–12.

[B32] Ortiz-Castro R. López-Bucio J. S. López-Bucio J. (2017). “ Physiological and molecular mechanisms of bacterial phytostimulation,” in Advances in PGPR Research. Wallingford: CABI. Eds. Singh H. B. Sarma B. K. Keswani C. , 16–28.

[B33] Raaijmakers J. M. Vlami M. De Souza J. T. (2002). Antibiotic production by bacterial biocontrol agents. Antonie van Leeuwenhoek 81, 537–547. doi: 10.1023/a:1020501420831 12448749

[B34] Ramírez‐Gil J. G. Castañeda‐Sánchez D. A. Morales‐Osorio J. G. (2017). Production of avocado trees infected with Phytophthora cinnamomi under different management regimes. Plant Pathol. 66, 623–632. doi: 10.1111/ppa.12620

[B35] Ramírez-Gil J. G. Morales-Osorio J. G. (2020a). Development and validation of severity scales of avocado wilt complex caused by Phytophthora cinnamomi, Verticillium dahliae and hypoxia anoxia disorder and their physiological responses in avocado plants. Agronomía Colombiana 38(1), 85–100. doi: 10.15446/agron.colomb.v38n1.78527

[B36] Ramírez-Gil J. G. Morales-Osorio J. G. (2020b). Integrated proposal for management of root rot caused by Phytophthora cinnamomi in avocado cv. Hass crops. Crop Prot. 137, 105271. doi: 10.1016/j.cropro.2020.105271 38826717

[B37] Ramírez-Gil J. G. Osorio J. G. M. (2021). Source of inoculum of pathogens, the origin of disorders and diseases management in avocado nurseries. Australas. Plant Pathol. 50, 457–468. doi: 10.1007/s13313-021-00796-y 30311153

[B38] Ramírez R S. Arias M J. D. Bedoya J. C. Rueda L E. A. Sánchez C. Y. Granada G S. D. (2015). Metabolites produced by antagonistic microbes inhibit the principal avocado pathogens *in vitro*. Agronomía Colombiana 33, 58–63. doi: 10.15446/agron.colomb.v33n1.48241

[B39] Rodríguez-Gálvez E. Haro-Diaz C. Maza-Aguirre S. Canahuire-Castillo F. Sullón-Saucedo J. Deising H. B. (2025). Lasiodiplodia theobromae disease symptom development in young avocado (Persea americana L.) plants depends on the inoculation method. Trop. Plant Pathol. 50, 18. doi: 10.1007/s40858-025-00700-9

[B40] Rodríguez-Gálvez E. Hilário S. Batista E. Lopes A. Alves A. (2021). Lasiodiplodia species associated with dieback of avocado in the coastal area of Peru. Eur. J. Plant Pathol. 161, 219–232. doi: 10.1007/s10658-021-02317-5

[B41] Rodríguez-Molina M. C. Santiago R. Blanco A. Pozo J. D. Colino M. I. Palo E. J. . (2003). Detección de Phytophthora cinnamomi en dehesas de Extremadura afectadas por “seca” y su comportamiento *in vitro*. Boletín Sanidad Vegetal Plagas 29, 627–640.

[B42] Salvatore M. M. Andolfi A. Nicoletti R. (2020). The thin line between pathogenicity and endophytism: The case of Lasiodiplodia theobromae. Agriculture 10, 488. doi: 10.3390/agriculture10100488 30654563

[B43] Sarbadhikary S. B. Mandal N. C. (2017). Field application of two plant growth promoting rhizobacteria with potent antifungal properties. Rhizosphere 3, 170–175. doi: 10.1016/j.rhisph.2017.04.014 38826717

[B44] Shahid I. Han J. Hardie D. Baig D. N. Malik K. A. Borchers C. H. . (2021). Profiling of antimicrobial metabolites of plant growth promoting Pseudomonas spp. isolated from different plant hosts. 3 Biotech. 11, 1–14. doi: 10.1007/s13205-020-02585-8 33489669 PMC7801537

[B45] SIEA (2024). Agricultural Export Statistics of Peru (Lima, Peru: Ministerio de Desarrollo Agrario y Riego del Perú). Available online at: https://app.powerbi.com/view?r=eyJrIjoiOGFjZGUwN2MtMzBkOS00YjEzLTg0NjEtODA1OTJiNzM0YjNiIiwidCI6IjdmMDg0NjI3LTdmNDAtNDg3OS04OTE3LTk0Yjg2ZmQzNWYzZiJ9 (Accessed June 20, 2025).

[B46] Singh M. Awasthi A. Soni S. K. Singh R. Verma R. K. Kalra A. (2015). Complementarity among plant growth promoting traits in rhizospheric bacterial communities promotes plant growth. Sci. Rep. 5, 15500. doi: 10.1038/srep15500 26503744 PMC4621411

[B47] Solís-García I. A. Ceballos-Luna O. Cortazar-Murillo E. M. Desgarennes D. Garay-Serrano E. Patiño-Conde V. . (2021). Phytophthora root rot modifies the composition of the avocado rhizosphere microbiome and increases the abundance of opportunistic fungal pathogens. Front. Microbiol. 11, 574110. doi: 10.3389/fmicb.2020.574110 33510714 PMC7835518

[B48] Van den Berg N. Christie J. B. Aveling T. A. S. Engelbrecht J. (2018). Callose and β‐1, 3‐glucanase inhibit Phytophthora cinnamomi in a resistant avocado rootstock. Plant Pathol. 67, 1150–1160. doi: 10.1111/ppa.12823

[B49] Van den Berg N. Swart V. Backer R. Fick A. Wienk R. Engelbrecht J. . (2021). Advances in understanding defense mechanisms in Persea americana against Phytophthora cinnamomi. Front. Plant Sci. 12, 636339. doi: 10.3389/fpls.2021.636339 33747014 PMC7971113

[B50] Weller D. M. (2007). Pseudomonas biocontrol agents of soilborne pathogens: looking back over 30 years. Phytopathology 97, 250–256. doi: 10.1094/PHYTO-97-2-0250 18944383

[B51] Zamioudis C. Mastranesti P. Dhonukshe P. Blilou I. Pieterse C. M. (2013). Unraveling root developmental programs initiated by beneficial Pseudomonas spp. bacteria. Plant Physiol. 162, 304–318. doi: 10.1104/pp.112.212597 23542149 PMC3641211

[B52] Zhang Y. Kong W. L. Wu X. Q. Li P. S. (2022). Inhibitory effects of phenazine compounds and volatile organic compounds produced by Pseudomonas aurantiaca ST-TJ4 against Phytophthora cinnamomi. Phytopathology® 112, 1867–1876. doi: 10.1094/phyto-10-21-0442-r 35263163

